# Extracellular Vesicle-derived circular RNAs confers chemoresistance in Colorectal cancer

**DOI:** 10.1038/s41598-019-53063-y

**Published:** 2019-11-11

**Authors:** Kha Wai Hon, Nurul Syakima Ab-Mutalib, Nik Muhd Aslan Abdullah, Rahman Jamal, Nadiah Abu

**Affiliations:** 1 UKM Medical Molecular Biology Institute (UMBI), Universiti Kebangsaan Malaysia, Kuala Lumpur, Malaysia; 2 Department of Oncology and Radiotherapy, UKM Medical Center, Universiti Kebangsaan Malaysia, Kuala Lumpur, Malaysia

**Keywords:** Cancer, Tumour biomarkers

## Abstract

Chemo-resistance is associated with poor prognosis in colorectal cancer (CRC), with the absence of early biomarker. Exosomes are microvesicles released by body cells for intercellular communication. Circular RNAs (circRNAs) are non-coding RNAs with covalently closed loops and enriched in exosomes. Crosstalk between circRNAs in exosomes and chemo-resistance in CRC remains unknown. This research aims to identify exosomal circRNAs associated with FOLFOX-resistance in CRC. FOLFOX-resistant HCT116 CRC cells (HCT116-R) were generated from parental HCT116 cells (HCT116-P) using periodic drug induction. Exosomes were characterized using transmission electron microscopy (TEM), Zetasizer and Western blot. Our exosomes were translucent cup-shaped structures under TEM with differential expression of TSG101, CD9, and CD63. We performed circRNAs microarray using exosomal RNAs from HCT116-R and HCT116-P cells. We validated our microarray data using serum samples. We performed drug sensitivity assay and cell cycle analysis to characterize selected circRNA after siRNA-knockdown. Using fold change >2 and p < 0.05, we identified 105 significantly upregulated and 34 downregulated circRNAs in HCT116-R exosomes. Knockdown of circ_0000338 improved the chemo-resistance of CRC cells. We have proposed that circ_0000338 may have dual regulatory roles in chemo-resistant CRC. Exosomal circ_0000338 could be a potential biomarker for further validation in CRC.

## Introduction

Colorectal cancer (CRC) is one of the most frequently diagnosed cancer worldwide and the second leading cause of cancer-related deaths^1^. In Malaysia, CRC is the second most commonly diagnosed cancer among males and the third among females between 2008 to 2013^2^. Chemotherapy is recommended for treatment of stage III and IV CRC patients^2^. Meanwhile, 5-fluorouracil (5-FU) is the main component of many regimens available for CRC patients, including FOLFOX and FOLFIRI^3,4^. However, researchers have estimated that nearly half of CRC patients develop resistance towards 5FU-based chemotherapy at a later stage of treatment, resulting in a high risk of cancer recurrence and mortality^3,4^. Absence of suitable clinical biomarker has become a major challenge for early prediction of chemoresistance among CRC patients^5^. Discovery of a novel biomarker is essential to address this issue for better management of CRC patients.

Exosomes have attracted increasing interest from the research community in recent years, especially as potential cancer biomarkers. Exosomes are a class of extracellular nanovesicles commonly ranging from 30 to 100 nm and secreted by all mammalian cells into body fluids^6^. These phospholipid bilayers cup-shaped nanovesicles carry nucleic acids, proteins, and lipids that reflect genetic information and physiological condition of originating cells^6^. Exosomes function as intercellular messengers to exchange biological molecules between cells, thus they play important roles in biological processes such as cellular metabolism and signalling^7^. Cancer cells have been shown to secrete exosomes actively with high abundance in the systemic circulation of cancer patients, therefore tumor-derived exosomes can be easily retrieved from liquid biopsies for cancer screening^8,9^.

Among different species of RNAs carried by tumor-derived exosomes, circular RNAs (circRNAs) are classified under a subset of endogenous non-coding RNAs with a size of >200 nucleotides^10^. CircRNAs are formed in a back-splicing event when a downstream 5′ splice site is joined to an upstream 3′ splice site to generate a circular loop structure which is superior against enzymatic degradation^11^. CircRNAs are more stable, abundant and highly conserved compared to their linear counterparts^12,13^. Certain circRNAs have been reported to function as miRNA sponges as well as interact with RNA-binding proteins (RBPs) to regulate post-transcriptional gene expression and mRNA translation^10^. CircRNAs have been widely investigated as potential biomarkers in CRC^14^. However, there is a limited understanding of the expression of circRNAs in chemoresistant CRC. Previously, we have reported on the profile of circRNA in drug-resistant HCT116 cells with clinical validation of candidate circRNAs in 25 FFPE (formalin-fixed paraffin-embedded) tissue samples from CRC patients^15^. To date, nothing has been reported on the analysis of circRNA expression in exosomes derived from chemoresistant CRC cells. Therefore, this study aims to generate the expression profile of circRNAs in exosomes derived from FOLFOX-resistant CRC cells. Differentially expressed exosomal circRNAs may serve as potential biomarker or target for early diagnosis of chemoresistance among CRC patients.

## Methods

### Cell culture

Human CRC cell line HCT-116 was obtained from the American Type Culture Collection (ATCC, USA). Cells were maintained in RPMI-1640 medium (R6504, Sigma-Aldrich, USA), supplemented with 2.0 g/L of sodium bicarbonate (Nacalai Tesque, Japan), 10% fetal bovine serum (FBS) (GE Healthcare, USA) and 1% penicillin-streptomycin solution (Nacalai Tesque Inc., Japan). Cells were maintained in a humidified New Brunswick CO_2_ Incubator (Eppendorf AG, Germany) at 37 °C under 95% air and 5% carbon dioxide. The medium was changed three times a week, and cells were passaged using 0.05% trypsin/EDTA solution (Nacalai Tesque Inc, Japan). Cell line authentication was performed using short tandem repeat (STR) profiling. Cells were routinely tested for mycoplasma contamination.

### Sample collection and ethics statement

All the serum samples used in this research were collected with complete informed consent from the participants at Oncology Ward and Oncology Clinic, UKM Medical Centre (UKMMC). Ethics approval was obtained from the Universiti Kebangsaan Malaysia Medical Research Ethics Committee before this study. All the sample collection was performed in accordance with relevant guidelines set by the Ethics Committee. CRC patients who had undergone at least half of FOLFOX/XELOX treatment cycles with no previous history of cancer were eligible for this study. Assessment of patient’s response towards FOLFOX/XELOX was evaluated by clinical oncologist based on the Response Evaluation Criteria In Solid Tumors criteria (RECIST 1.1)^16^. A total of 17 CRC patients were recruited: 10 FOLFOX-resistant and 7 FOLFOX-sensitive (Supplementary Material S1). Patient’s serum was filtered through a 0.22-μm syringe for pre-processing. Exosomal RNA was extracted from filtered serum using QIAGEN exoRNeasy Midi Kit (QIAGEN GmbH, Germany) according to manufacturer protocol. Final exosomal RNA elution in 14 μL of RNase-free water was kept at −80 °C for downstream applications.

### Development of folfox-resistant HCT116 cells (HCT116-R)

FOLFOX-resistant CRC cells were generated by exposing parental HCT-116 cells (HCT116-P) to FOLFOX at clinically relevant doses according to the planned induction schedule, with reference to the previous literature^17^. 5-Fluorouracil (5-FU) and oxaliplatin were purchased from Acros Organics (New Jersey, USA). Drug induction schedule was planned for 10 cycles; each cycle lasted for two weeks. In each cycle, the cells were exposed to 25 μM of 5-FU and 0.625 μM of oxaliplatin for 48 hours. The surviving cells were cultured in drug-free normal medium for the remaining 12 days. Finally, the HCT116 FOLFOX-resistant cells (HCT116-R) were maintained in complete culture medium containing a low dose of FOLFOX (3.125 μM of 5-FU + 0.625 μM of oxaliplatin). These cell lines were previously developed by our lab^15^.

### Drug sensitivity assay

Inhibition of cell growth between HCT116-P and HCT116-R cell lines towards 5-FU, oxaliplatin and FOLFOX respectively was assessed by 3-(4,5-dimethylthiazol-2yl)-2, 5-diphenyltetrazolium bromide (MTT) (Acros Organics, USA). 5 × 10^3^ cells/well were seeded into 96-well plates and incubated for 24 hours. Another 48-hours incubation was performed in presence or absence (control) of different concentrations of 5-FU, oxaliplatin, and FOLFOX. Upon the end of incubation, the MTT solution was added to each well and incubated for an additional three hours. Formazan crystals were dissolved in DMSO (Fisher Scientific, USA). The intensity of color development was measured at a wavelength of 570 nm using a microplate reader (Thermo Scientific, USA). Absorbance values were compared with corresponding controls to calculate relative cell viability and drug resistance index of each cell line. All the experiments were performed in triplicates.

### Isolation of exosomes from cell culture medium

Ultracentrifugation procedure was modified based on the previous protocol by Thery *et al*.^18^. Cells were cultured in culture medium supplemented with 10% exosome-free FBS for 72 hours. Used culture medium was collected, pre-cleared and filtered through a 0.22-μm syringe filter. The filtered medium was subjected to stepwise ultracentrifugation using Optima™ XE-100 ultracentrifuge (Beckman Coulter, USA) to a final speed of 100,000 × g (4 °C, overnight). The supernatant was discarded while the fraction was resuspended in sterile PBS and spun again 100,000 × g (4 °C, overnight) to purify exosomes. Lastly, exosomes were collected in PBS as an exosomes-enriched fraction.

### Dynamic light scattering and zeta potential analysis

Particle size measurement and zeta potential analysis of exosomes were performed using Zetasizer Nano ZS system (Malvern Instruments, Malvern, U.K.) equipped with a 633-nm He–Ne laser and operating at an angle of 173°. Exosomes were diluted 1: 100 in sterile PBS to a total volume of 1 mL to be loaded into a disposable cuvette for particle size measurement. For zeta potential analysis, exosomes were diluted 1:100 in ultrapure water and loaded into Malvern Folded Capillary Zeta Cell (model no: DTS1070). Data were acquired and analyzed using Zetasizer Software (V7.03) (Malvern Instruments). All the experiments were performed in triplicates.

### Transmission electron microscopy

Purified exosomes were diluted to 1:1000 in PBS. Five microliters of diluted exosomes were dropped on Formvar-carbon coated EM grids and left aside to allow membranes adsorb for 20 minutes. The vesicles-coat grids were fixed with 0.6% glutaraldehyde for four minutes and washed twice with distilled water for one minute each. The grids were stained with 2% uranyl acetate at pH 7 for 5 minutes. Finally, the grids were viewed using a Philips CM12 transmission electron microscope (Philips, The Netherlands) at a voltage of 80 kV. Digital images with scale bar provided were captured at a magnification of 10,000–200,000.

### Western blot

Exosome lysate was prepared by adding RIPA buffer directly to an exosomes-enriched fraction in PBS and incubated on an orbital shaker at 4 °C for 45 minutes. The mixture was centrifuged, and the supernatant was collected as protein lysate. Protein lysate was quantified using Bradford assay. Fifty μg of proteins were resolved on SDS-poly acrylamide gel electrophoresis (SDS-PAGE) and transferred to nitrocellulose membrane. The membrane was blocked with 5% skimmed milk in TBS-T before incubated with primary antibodies at 4 °C overnight. Primary antibodies used were mouse monoclonal anti-human TSG101 (Cat# NB200-112 Novus Biologicals, USA. 1:1000 dilution), rabbit polyclonal anti-human CD9 (Cat# ab92726; Abcam, UK. 1: 1000 dilution) and mouse monoclonal anti-human CD63 (Cat# Sc-5275 Santa Cruz Biotechnology Inc., USA. 1:1000 dilution). The membrane was washed with TBS-T before incubated with HRP-conjugated secondary antibodies at room temperature for 1 hour. Finally, the blot was washed with TBS-T three times and incubated with Pierce ECL Western Blotting Substrate (Life Technologies, USA) for 10 minutes. Protein bands were visualized via chemiluminescence using ChemiDoc MP Imaging System (Bio-Rad, USA).

### Exosomes uptake assay

HCT116-R exosomes were labeled with a red fluorescent dye PKH26 (Sigma-Aldrich, USA), according to the protocol previously reported^19^. Briefly, 300 μL of exosomes were resuspended into 100 μL of Diluent C while another 350 μL of Diluent C was mixed with 1.4 μL of PKH26 to prepare staining solution. Exosome suspension was mixed with a staining solution and incubated for 5 minutes. An equal volume of 1% bovine serum albumin (BSA) was added into the mixture to stop the reaction. Labeled exosomes were added to HCT116-P cells and incubated at 37 °C for 6 hours. The cells were fixed with 4% paraformaldehyde, counter-stained with DAPI and visualized under fluorescence microscopy using DeltaVision deconvolution microscope (Applied Precision, USA). Images were deconvolved using SoftWoRx Suite v.3.5.1. (Applied Precision).

### Co-culture assay

HCT116-R cells were seeded into 1-μm porous Transwell inserts (Corning, USA) as the upper chamber hanging over 6-well plate. Lower chamber well was plated with HCT116-P cells. The ratio of cells in the upper chamber to cells in the lower chamber was 4:1 (HCT116-R: HCT116-P). In control wells, both chambers were plated with HCT116-P cells at the same ratio of 4:1. After 72 hours of co-culture, half of the HCT116-P cells in the lower chamber was harvested for qPCR analysis and another half for drug sensitivity assay using MTT. All the experiments were performed in triplicates.

### Isolation of exosomal RNA and QC

QIAGEN exoEasy Maxi Kit (QIAGEN GmbH, Germany) coupled with QIAGEN miRNeasy Mini Kit (QIAGEN GmbH, Germany) was used to isolate exosomal RNA from cell culture media. Once the exosomes were bound to the exoEasy Maxi spin column membrane and washed with buffer XWP, 700 μL of QIAzol Lysis Reagent (QIAGEN GmbH, Germany) was added directly onto the membrane and centrifuged to collect lysate. The lysate was proceeded with the miRNeasy Mini Kit by following the manufacturer’s protocol. Finally, 14 μL of RNase-free water was used to elute the RNAs from the column. Eluted RNAs was stored at −80 °C. The RNA concentration was quantified using NanoDrop 2000 Spectrophotometer (Thermo Scientific, USA). RNA quantification and profiling were performed using on-chip-electrophoresis via Agilent 2100 Bioanalyzer and RNA 6000 Pico kit. (Agilent Technologies, USA).

### Circular RNA microarray

Total RNAs were digested with RNase R (Epicenter, Inc., USA) to remove linear RNAs and enrich circRNAs. Enriched circRNAs were amplified and transcribed into fluorescent cRNA using a random priming method (Arraystar Super RNA Labeling Kit; Arraystar, USA). Labeled cRNAs were hybridized onto Arraystar Human circRNA Array V2 (8 × 15 K, Arraystar, USA). After having washed the slides, the arrays were scanned by the Agilent G2565CA Microarray Scanner System (Agilent Technologies, USA). Agilent Feature Extraction software (version 11.0.1.1) was used to analyze acquired array images. Quantile normalization and subsequent data processing were performed using Arraystar software. Differentially expressed circRNAs with statistical significance of p < 0.05 and fold change >2 between two groups were identified through Volcano Plot filtering. Hierarchical clustering was performed to display distinguishable circRNAs expression pattern among samples.

### Annotation of circRNA-miRNA interaction

Arraystar’s in-house miRNA target prediction software (Arraystar, USA) based on TargetScan^20^ and Miranda algorithms^21^ were used to predict miRNA response elements (MREs) and putative circRNA/miRNA interactions for all differentially expressed circRNAs, based on seed-sequence complementarity. The following parameters were used for this Arraystar MRE analysis: seed type (seed-sequence complementarity) between nucleotides 2–7, the proximity of AU-richness to seed sequence, and proximity to nucleotides pairing with the miRNA’s 3 pairing sequence (nucleotides 13–16). Targeted miRNAs were ranked according to their mirSVR scores, and five miRNAs with the highest mirSVR score were identified as MREs for each circRNAs.

### Prediction of cancer-related circRNA-miRNA-target gene associations

We combined the analysis from TargetScan (http://www.targetscan.org)^20^ and miRDB (http://www.mirdb.org)^22^ to predict target genes of miRNAs/MREs for further analysis of their functions. Predicted gene functions in the networks were annotated in ToppGene (https://toppgene.cchmc.org)^23^ using Gene Ontology (GO) enrichment analysis (www.geneontology.org/)^24^ and Kyoto Encyclopedia of Genes and Genomes (KEGG) pathway analysis (www.genome.jp/kegg/)^25^.

### Validation of candidate circRNAs using qRT-PCR

Quantitative reverse transcription PCR (qRT-PCR) was used to validate selected circRNAs from microarray data in clinical samples. Five µg of RNA from each sample was subjected to reverse transcription (RT) with random hexamers using RevertAid RT Kit (Thermo Scientific), according to manufacturer’s instructions. Divergent primers (instead of commonly used convergent primers) were designed and optimized for four selected circRNAs based on the sequences acquired from the database “circBase” (http://circbase.mdc-berlin.de)^26^. Glyceraldehyde 3-phosphate dehydrogenase (GAPDH) and ribosomal protein L13 (RPL13) were used as housekeeping genes. Sequences for all the primers used in this study were tabulated in Supplementary Material S3. Target specificity of PCR primers was verified using BLAST (http://blast.ncbi.nlm.nih.gov/Blast.cgi)^27^ and Sanger sequencing (Supplementary Material S4). The real-time PCR assay was carried out using SensiFast™ SYBR No-ROX reagents (Bioline Reagent, Ltd., UK) and CFX96 Touch™ Real-Time PCR Detection System (Bio-Rad, USA). The appearance of a single peak in the melting curve indicated the specificity of PCR results. All the experiments were performed in triplicates. Data were analyzed using the ΔCt method, 2^−ΔΔCT^, to represent relative expression levels of circRNAs.

### Loss-of-function by small interfering RNA (siRNA)

Loss-of-function was simulated using siRNAs purchased from Integrated DNA Technologies targeting hsa_circ_0000338 gene transcript. Forty nM of siRNAs were transfected into HCT116-R cells using Lipofectamine 2000 (Thermo Fisher Scientific, USA) according to manufacturer protocol. Drug sensitivity assay and cell cycle analysis were performed to compare between transfected HCT116-R cells and control HCT116-R cells. All the experiments were performed in triplicates.

### Statistical analysis

Statistical analysis for cell-based assays, functional studies, and microarray validation was performed using GraphPad Prism version 7.00 for Windows (GraphPad Software, USA). Two-tailed Student’s t-test was applied to calculate the statistical significance of the difference between two independent groups (p < 0.05). Student’s t-test and fold change values were used to analyze the statistical significance of the microarray results for the respective comparison of two groups. The false discovery rate (FDR) was determined to calculate the adjusted *p-value*s based on the Benjamini-Hochberg procedure, which controls the number of false-positive in multiple testing of microarray data for a more accurate analysis^28^. The threshold value used to designate differentially expressed circRNAs in microarray was set at a fold change of ≥2.0 or ≤0.5 (p < 0.05). All the data were presented as mean ± SD (*p < 0.05, **p < 0.01, ***p < 0.001) unless otherwise stated.

### Ethical conduct of research

The authors state that they have obtained appropriate institutional review board approval from UKM Medical Centre (UKMMC) for all human experimental investigations. Also, informed consent has been obtained from the participants involved.

## Results

### Establishment of drug-resistant HCT116 human colorectal cells (HCT116-R)

Based on Table 1, HCT116-R cells were significantly 5.87 times more resistant to 5-FU and 2.58 times more resistant to oxaliplatin as compared to HCT116-P cells (p < 0.001). As compared to HCT116-P cells, HCT116-R cells were between 3.27 to 107.44 times more resistant to the two different combinations of FOLFOX (p < 0.05) (Table 1).

**Table 1:** Drug sensitivity indices for parental HCT116-P and resistant HCT116-R cell lines. Data were presented as mean ± SD (*p < 0.05, **p < 0.01, ***p < 0.001).

LD50 (µg/mL)	5-FU	Oxaliplatin	5-FU with 20 µg/mL oxaliplatin	Oxaliplatin with 120 µg/mL 5-FU
HCT116-R	40.64 ± 7.43	23.38 ± 4.48	22.62 ± 4.98	0.01 ± 0.02
HCT116-P	6.92 ± 1.72	9.06 ± 2.08	6.92 ± 1.72	0.0001182 ± 0.0004481
Drug-resistant index	5.87	2.58	3.27	107.44
P-value	≤0.001	≤0.001	≤0.001	0.01
Significance	***	***	***	*

The experiment was performed in triplicates.

### Characterization of exosomes from cell culture

**Figure 1:**
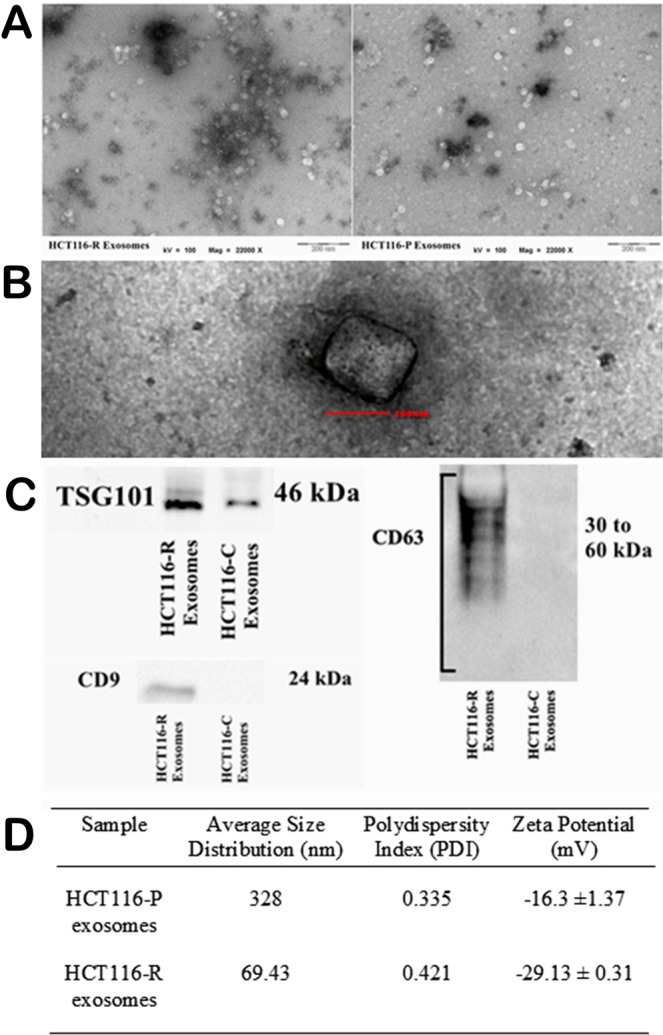
Characterization of exosomes. (**A**) Representative TEM images of exosomes at 22000x magnification (Left) HCT116-R exosomes and (Right) HCT116-P exosomes. (**B**) Enlarged planar view of a cup-shaped structure for single exosome in our samples at 87000x magnification. (**C**) Western blot analysis of exosomes revealed differential expression of TSG101, CD9, and CD63 between HCT116-R exosomes and HCT116-P exosomes. (**D**) Average size distribution and zeta potential of exosomes measured in Zetasizer.

TEM analysis confirms the presence of translucent, cup-shaped vesicles, corresponding to the unique characteristics of exosomes (Fig. 1A,B). For Western blot analysis, we detected TSG101 in both HCT116-P and HCT116-R exosomes (Fig. 1C). CD9 and CD63 were only visibly expressed in HCT116-R exosomes whereas faintly expressed in HCT1‘16-P exosomes (Fig. 1C). We used Zetasizer to measure average size distribution and zeta potential of our exosomes as the physical properties of our exosomes, according to the guidelines by the International Society for Extracellular Vesicles (ISEV). The average size of HCT116-P exosomes was 300.7 nm with a polydispersity index (PDI) of 0.288 while HCT116-R exosomes were 69.43 nm with PDI of 0.421 (Fig. 1D). PDI values represent size ranges of particles present in the solution. PdI values for both cell line exosomes were moderately high between 0.288 to 0.421, indicating a relatively multimodal particle size distribution due to the natural attribute of exosomes being secreted in variable sizes^29^. Zeta potential of exosomes refers to the electric potential difference between the stationary layer of ions bound to the vesicles and solution^30^. Average zeta potential for HCT116-P exosomes was −16.3 mV ± 7.92 mV, as compared to HCT116-R exosomes on average of −29.13 mV ± 0.31 mV (Fig. 1D).

### Circular RNA Microarray Profiling

**Figure 2:**
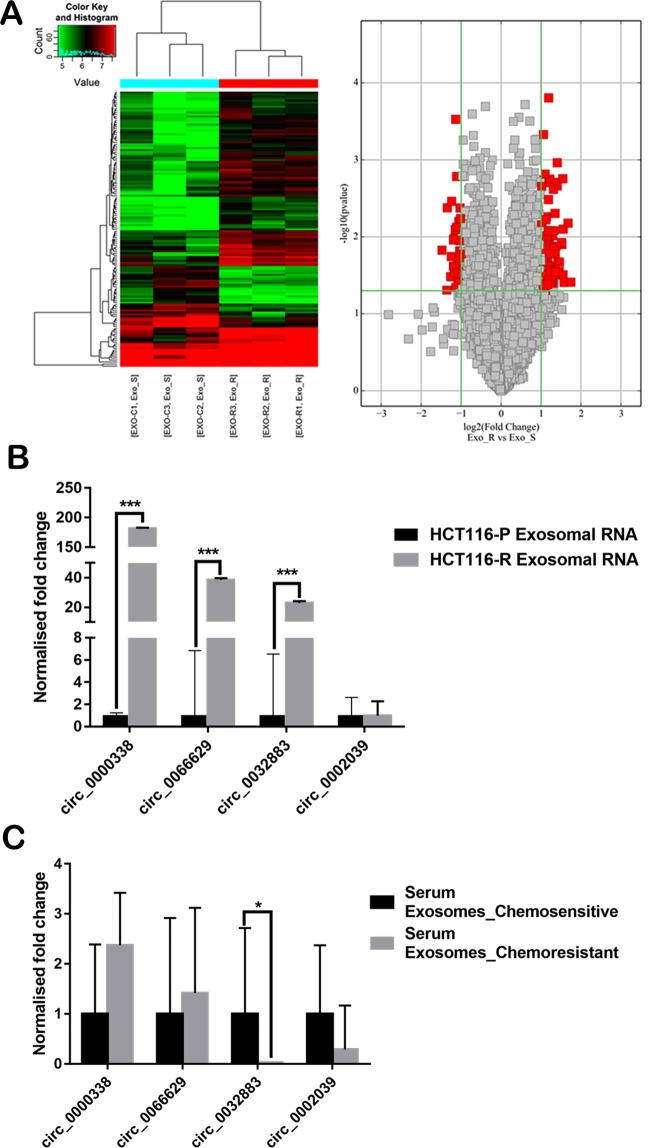
(**A**) Hierarchical clustering and (**B**) volcano plot used to identify differentially expressed circRNAs between exosomes. (Fold change >2, p < 0.05). RT-qPCR validation of four selected circRNAs on (**C**) cell line exosomes (**D**) serum exosomes. Data were presented as mean ± SD (*p < 0.05, **p < 0.01 and ***p < 0.001). The experiment was performed in triplicates.

Based on our microarray analysis, 139 circRNAs were differentially expressed between HCT116-R exosomes and HCT116-P exosomes (fold change > 2, p < 0.05), in which 105 circRNAs were upregulated with another 34 circRNAs downregulated in HCT116-R exosomes (Fig. 2).

### Validation of candidate circRNAs

Among 105 differentially upregulated circRNAs, we selected 4 circRNAs (hsa_circ_0032883, hsa_circ_0066629, hsa_circ_0002039 and hsa_circ_0000338) for validation (Table 2). We validated the selected circRNAs in our cell line exosomes, in which hsa_circ_0000338, hsa_circ_0032883, and hsa_circ_0066629 were significantly upregulated in HCT116-R exosomes as compared to HCT116-P exosomes (Fig. 2B). We also investigated the expression of these selected circRNAs in 17 clinical samples (7 responders and 10 non-responders). Interestingly, hsa_circ_0032883 was significantly downregulated in serum exosomes of non-responder patients as compared to responder patients (Fig. 2C). Based on our qRT-PCR findings, we predicted that hsa_circ_0000338 could be a potential target in regulating chemo-resistance.

**Table 2:** Information of 4 selected circRNAs for validation.

CircRNA ID	Type	Chrom	Gene Symbol	Strand	p-value	FDR	Fold change
hsa_circ_0032883	exonic	chr14	EML5	−	1.96E-03	0.43	2.58
hsa_circ_0066629	exonic	chr3	DCBLD2	−	2.70E-02	0.45	2.38
hsa_circ_0000338	exonic	chr11	FCHSD2	−	3.61E-02	0.45	2.25
hsa_circ_0002039	exonic	chr1	OSBPL9	+	6.62E-03	0.43	3.18

Chrom: Chromosome; FDR: False discovery rate.

### Prediction of circRNA–miRNA–mRNA associations and bioinformatics analysis

**Figure 3:**
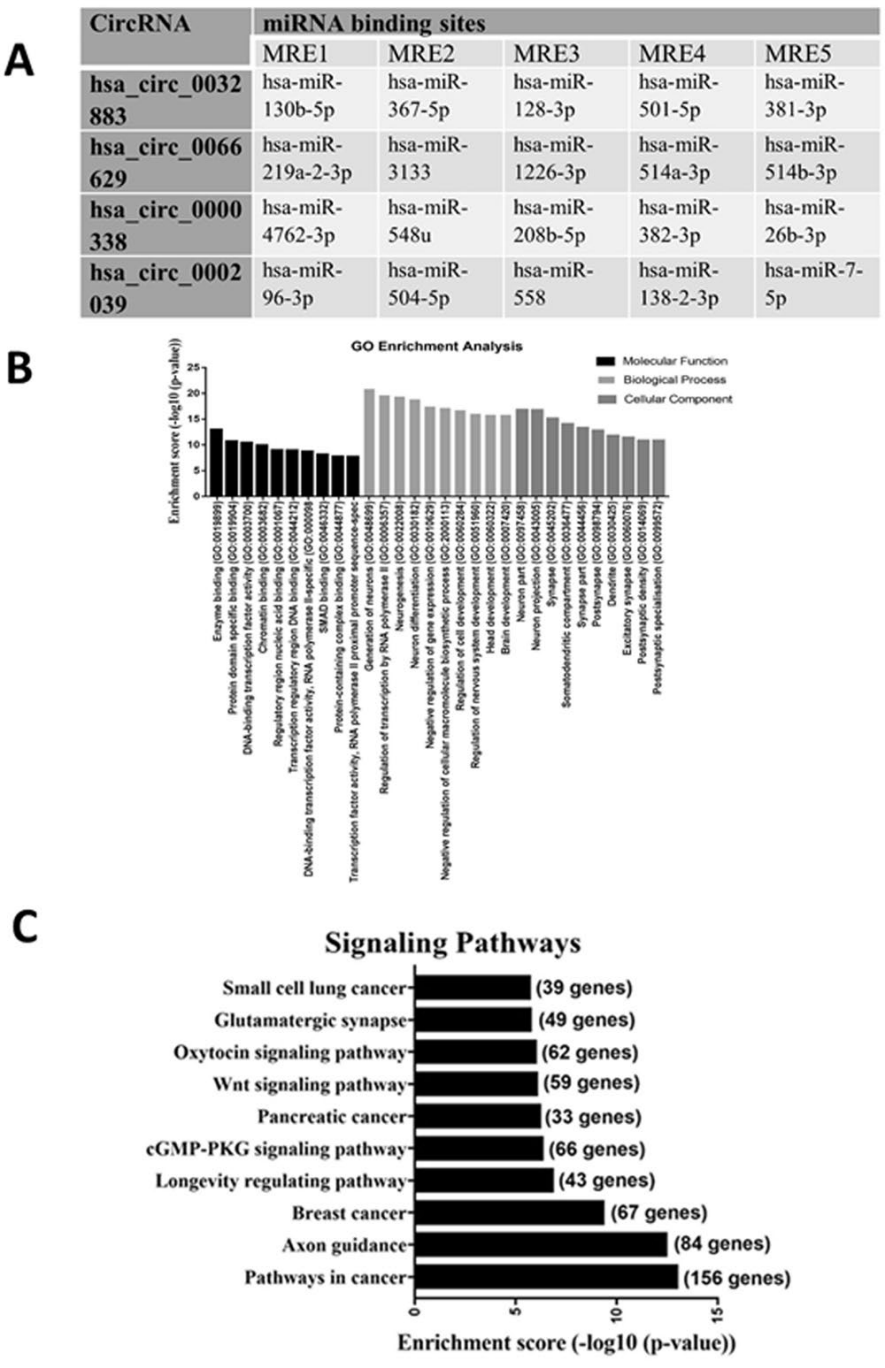
(**A**) miRNA binding sites prediction (MREs) of upregulated circRNAs. (**B**) GO enrichment analysis of target genes related to upregulated circRNAs. (C) KEGG pathway analysis of target genes.

To elucidate the molecular mechanism of circRNAs, we analyzed the miRNA response elements (MREs) associated with upregulated circRNAs to identify any circRNA–miRNA connections based on TargetScan and miRanda. Targeted miRNAs were ranked according to their mirSVR scores, and five miRNAs with the highest mirSVR score were identified as MREs for each circRNAs (Fig. 3A). GO enrichment analysis revealed several target genes of upregulated circRNAs contribute towards the regulation of transcription and cell development, as well as negative regulation of biosynthesis and gene expression (Fig. 3B). KEGG analysis reported the top ten pathways associated with upregulated circRNAs, including longevity regulating pathway, Wnt signaling pathway, cGMP-PKG signaling pathway and different pathways in cancer (Fig. 3C).

### Exosomes mediate transfer of circRNAs to adjacent or distant cells for intercellular communication

**Figure 4:**
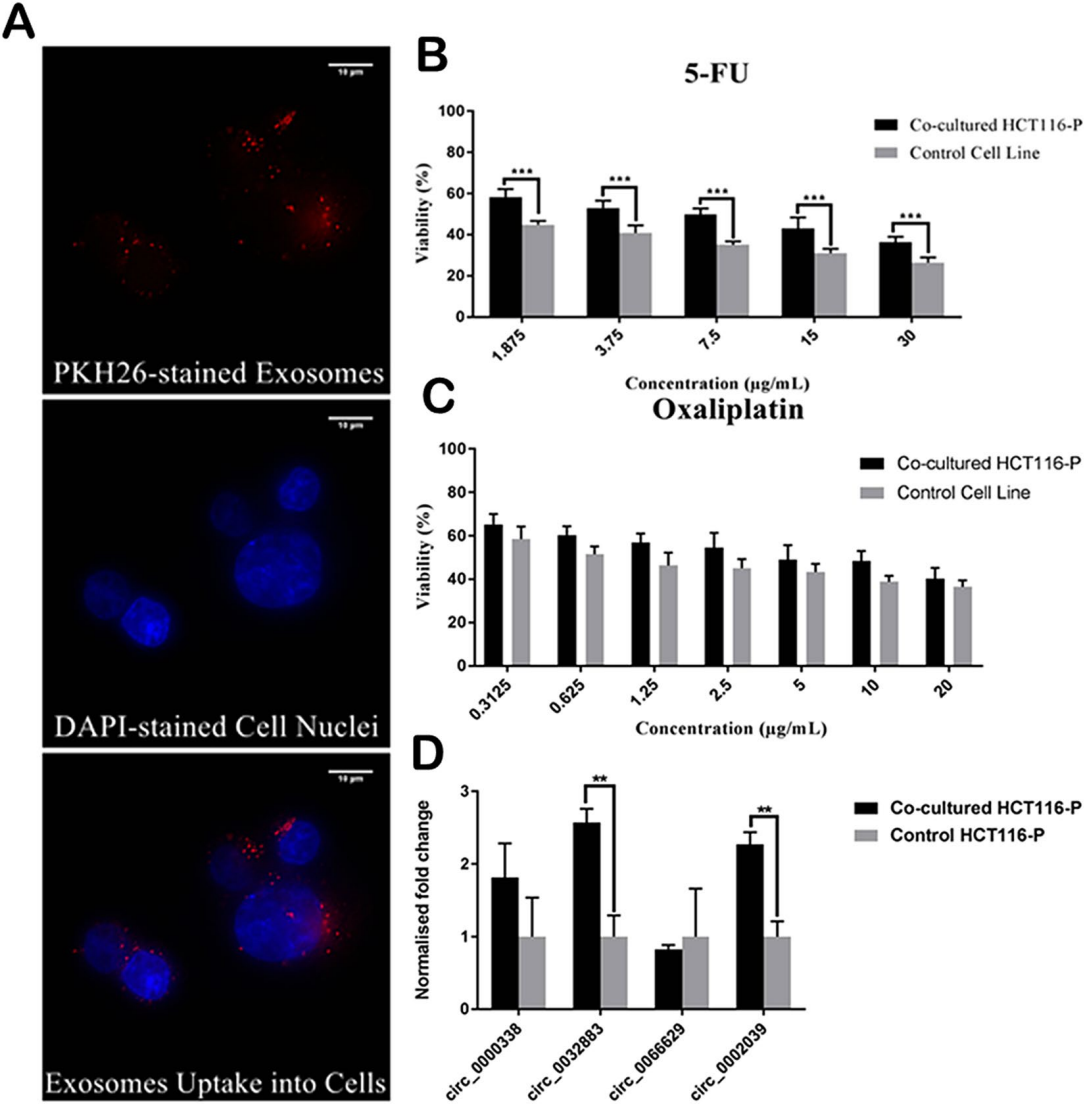
Exosomes uptake assay. (**A**) Representative fluorescence microscopic images (60X magnification) of exosomes uptake assay. Scale bar: 10 µm. (Up) PKH26-stained exosomes. (Middle) Cell nuclei counterstained with DAPI. (Down) Merged image of exosomes and cell nuclei showed that HCT116-R exosomes were incorporated into HCT116-P cells. (**B**–**C**) Drug sensitivity assay between co-cultured HCT116-P cells and control HCT116-P cells using (**B**) 5-FU and (**C**) Oxaliplatin. (**D**) RT-qPCR validation of four selected circRNAs on co-cultured cells and control cells. Data were presented as mean ± SD (*p < 0.05, **p < 0.01, ***p < 0.001). The experiment was performed in triplicates.

We investigated whether chemoresistance could be transferred between CRC cells via exosomes. Our fluorescence microscopy visualized the uptake of PKH26-stained HCT116-R exosomes (red) into the cytoplasm of HCT116-P cells (Fig. 4A). We co-cultured HCT116-P cells with HCT116-R cells for 72 hours when compared with control HCT116-P cells in drug sensitivity assay. Our results showed that co-cultured HCT116-P cells had significantly higher viability in 5-FU as compared with control cells (Fig. 4B). We compared the expression of the 4 selected circRNAs between co-cultured HCT116-P cells and control HCT116-P cells using qRT-PCR analysis. Our results revealed that hsa_circ_0032883 and hsa_circ_0002039 were significantly upregulated in co-cultured HCT116-P cells as compared to control cells (Fig. 4D). This indicates that HCT116-R exosomes may perform the selective transfer of circRNAs into the recipient cells to confer chemoresistance.

### Functional analysis of circ_0000338 via loss-of-function in siRNA knockdown

**Figure 5:**
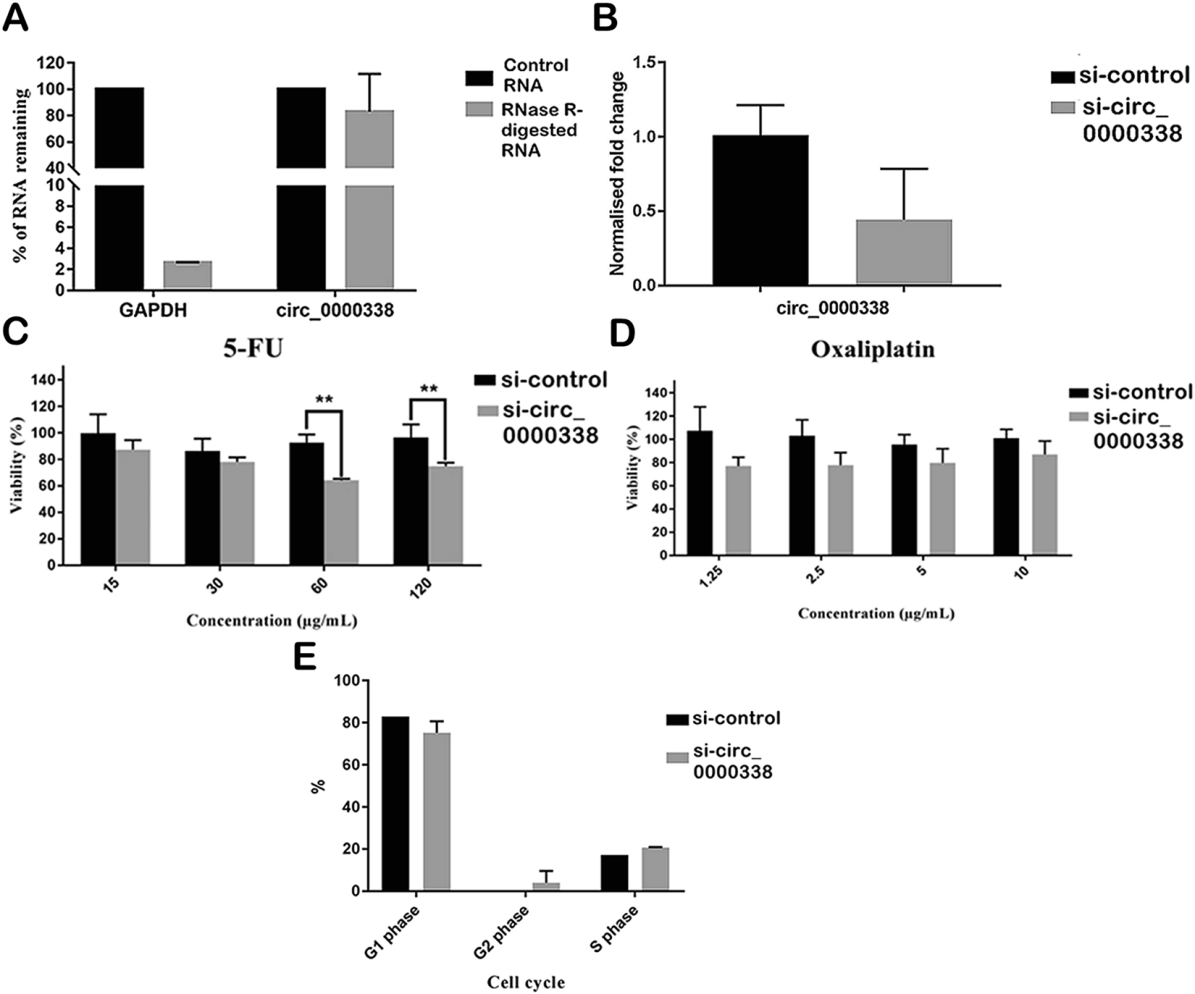
(**A**) Proof-of-concept. Enrichment of circ_0000338 after RNase R treatment as compared to linear RNA GAPDH. (**B**) Suppression of circ_0000338 after successful knockdown with siRNA transfection as validated by qRT-PCR. Si-control represents the HCT116-R cells with transfection control while si-circ_0000338 represents the HCT116-R cells transfected with siRNA targeting circ_0000338. (**C**,**D**) Drug sensitivity assay between transfected HCT116-R cells and control cells using (**C**) 5-FU and (**D**) Oxaliplatin. (**E**) Cell cycle analysis between transfected HCT116-R cells and control cells. Data were presented as mean ± SD (*p < 0.05, **p < 0.01, ***p < 0.001). Experiment was performed in triplicates.

Based on our qRT-PCR findings, we predicted that hsa_circ_0000338 could be a potential target in regulating chemo-resistance. As a proof-of-concept to confirm that junction sequence of our PCR amplicons is formed from the real back-splicing junction, our RNA sample was digested with RNase R to degrade linear RNAs and followed by qRT-PCR to compare expression on GAPDH and hsa_circ_0000338. As shown in Fig. 5A, GAPDH expression depleted to 2.7% of control reaction, while hsa_circ_0000338 maintained around 78% of control reaction. This shows that RNase R treatment degrades linear RNAs and enriches circRNAs simultaneously. We performed siRNA knockdown of hsa_circ_0000338 in transfected HCT116-R, in which the suppression of this circRNA was confirmed by qRT-PCR as shown in Fig. 5B. We compared transfected cells and control cells in drug sensitivity assay (Fig. 5C,D). Viability of transfected HCT116-R cells was significantly increased in 5-FU concentrations of 60 µg/mL and above upon the knockdown of hsa_circ_0000338. 5-FU and oxaliplatin are the cytotoxic drugs that interfere with DNA synthesis and promote apoptosis in CRC cells^31,32^. Transfected HCT116-R cells and control HCT116-R cells were compared in cell cycle analysis to measure apoptotic cell death upon the knockdown of hsa_circ_0000338 but no significance was reported (Fig. 5E).

## Discussion

Our study has reported on the profiling of circRNAs in exosomes isolated from FOLFOX-resistant HCT116-R cells. We selected the HCT116 cell line to develop a stable resistant clone based on drug sensitivity screening by comparing with other cell lines (data not shown). Literature review reveals the importance of HCT116 cell line as a well-characterized cell model for *in-vitro* studies of drug resistance in CRC^33^. In this study, the development and routine maintenance of HCT116-R cells were performed with reference to previous methodology^34,35^. Parental HCT116-P cells were constantly exposed to high drug concentrations over certain drug induction schedule with the surviving cells being sub-cultured. Our HCT116-R cells have shown significantly higher LD_50_ values to 5-FU and oxaliplatin as compared to parental cells. Our HCT116-R cells were maintained in medium containing low drug concentration to retain the drug resistant phenotype as suggested by previous studies^17,36^. We compared the parental cells and resistant cells in drug sensitivity assay periodically to confirm the preservation of drug resistance. Currently, drug sensitivity assay is the only approach to confirm drug resistance in cells by calculating the drug sensitivity indices as discussed in the previous literature^35^.

For the characterization of our cell line exosomes, we have referred to the guidelines by ISEV^37^. We have characterized our exosomes using TEM, DLS, zeta potential analysis and Western blot for comprehensive physical profiling of exosomes. Our DLS analysis has shown that HCT116-P exosomes have a larger average size distribution (328 nm) as compared to HCT116-R exosomes (69.43 nm). We speculate that the huge difference in the size distribution of our exosomes could be attributed to the limitations of DLS as well as the possible effect of acquired drug resistance. DLS provides reliable and reproducible results in the measurement of nanoparticles including exosomes, but this technique has limitations for single quantification of exosomes^38^. DLS does not visualize the particles individually but it calculates the hydrodynamic size of particles based on fluctuations in scattered light intensity caused by the Brownian movement of particles^39^. DLS measurement can be affected by the presence of large aggregates/molecules which scatter more light intensity^40^. Accuracy and precision of DLS are reduced in poly-dispersed samples like exosomes which are highly heterogeneous in size and shape^39^. Meanwhile, acquired drug resistance may alter the cellular mechanisms in HCT116 cells and subsequently affect the production of exosomes. TP53 status in HCT116 cells has been shown to interact with the ESCRT-dependent formation of exosomes to influence the size of exosomes being secreted^41^. HCT116 cells with wild-type TP53 produced bigger exosomes in terms of size (188 nm) as compared to those with mutant TP53 (107 nm) and null TP53 (80 nm)^41^. Further studies could be conducted to elucidate the effect of acquired drug resistance on exosomes production in CRC cells.

Zeta potential of exosomes refers to the electric potential difference between the stationary layer of ions bound to the vesicles and solution^30^. Our HCT116-R exosomes had a more negative zeta potential (−29.3 mV) as compared to HCT116-P exosomes (−16.3 mV). Zeta potential could range from −40 mV to 54 mV for exosomes isolated from various cancer and normal cells^38,42^. Previous studies have shown that the negative zeta potential of cancer-derived exosomes could be attributed to a large number of sialic acids present on the surface membrane of exosomes^43^. Researchers have proposed that zeta potential with an absolute value greater than −20 mV guarantees colloidal stability of exosomes to prevent aggregation^44^. Weakly negative zeta potential of HCT116-P exosomes may lead to the aggregate formation that gives rise to larger size distribution in DLS. Our wide field TEM images displayed multiple translucent vesicles in all our samples in addition to the enlarged view of a single cup-shaped vesicle, confirming the presence and shape of exosomes in our samples. In Western blot analysis, we evaluated 2 different classes of “exosome-enriched” proteins (cytosolic and transmembrane proteins) based on guidelines by ISEV^37^. All our samples expressed cytosolic protein TSG101, which could be strong evidence to show that our samples are most likely exosomes. CD9 and CD63 are present in HCT116-R exosomes but not HCT116-P exosomes. CD9 and CD63 are transmembrane proteins commonly detected in exosomes but could be absent in a certain subset of exosomes^6,37^. We also postulate that the high level of TSG101 in HCT116-R exosomes could be due to the increased expression of cellular TSG101. Previously it has been reported that colorectal cancer cell lines resistant to oxaliplatin expressed higher levels of mRNA and protein of TSG101^45^. However, our study did not further evaluate the other classes of co-isolated proteins (intracellular and extracellular proteins) to determine the purity of our exosomes as suggested by ISEV^37^.

Our microarray profiling revealed a total of 105 up- and 34 downregulated circRNAs in HCT116-R exosomes with fold change >2. Most of the differentially expressed circRNAs detected in our microarray are exonic circRNAs, which is the most abundant type of circRNAs^10^. Previous studies have mostly reported on the profiling of circRNAs in CRC samples (cell line/tissue/serum) with comparison to normal/healthy subjects^46–49^. There are only a few reports on the expression of circRNAs in chemo-resistant CRC. Xiong *et al*. performed circRNA microarray analysis on 5-FU resistant CRC cell line, in which 47 circRNAs were differentially upregulated and 24 downregulated^50^. Our team has also previously reported on the circRNA microarray profiling of drug-resistant HCT116 cells with clinical validation of candidate circRNAs in 25 FFPE samples from CRC patients via qPCR^15^. Similarly, little is known about the expression of exosomal circRNAs in chemo-resistant cancers. Xu *et al*. performed RNA-seq to identify a total of 275 differentially expressed circRNAs in extracellular vesicles (EVs) isolated from the serum of patients with endometrial cancer^51^. Wang *et al*. have investigated circRNA expression in exosomes derived from breast cancer cell lines using microarray analysis^52^. The same study has also reported on circRNA expression profile in serum of patients with metastatic breast cancer and localized breast cancer using RNA-seq approach^52^. Our current findings have provided a novel insight into the expression profile of circRNAs in CRC exosomes, which could be essential to engage further investigation on the roles of exosomal circRNAs in FOLFOX-resistant CRC.

We have selected four differentially upregulated circRNAs (hsa_circ_0032883, hsa_circ_0066629, hsa_circ_0002039, and hsa_circ_0000338) for validation and *in silico* analysis. Previously we have validated two of the circRNAs in tissue samples and discovered that hsa_circ_0032883 and hsa_circ_0000338 were upregulated in chemoresistant tissue^15^. We also performed the validation in EVs isolated from the cell lines, but the EVs were isolated using a precipitation-based method, whereas for this study, we used ultracentrifugation^15^. The regulation of the EVs were the same using both techniques. Our technical validation in cell line exosomes showed good consistency with our microarray data, with 3 out of 4 selected circRNAs (hsa_circ_0000338, hsa_circ_0032883, and hsa_circ_0066629) were significantly upregulated in HCT116-R exosomes. For our clinical validation, hsa_circ_0032883 was significantly downregulated in serum exosomes of chemo-resistant patients. Human serum contains a heterogenous mixture of exosomes of different cellular origins^53–55^. The circRNAs profiling in serum exosomes should be more complicated as compared to our analysis using pure cell line exosomes. Our microarray data generated using pure cell line exosomes did not fully complement the actual clinical investigation, probably due to the limited sample size in our clinical validation. We validated our microarray data in serum exosomes of CRC patients with different responses towards FOLFOX therapy. The difference in physiological profile among these CRC patients is predicted to be little. Larger sample size would be essential to have more statistical power in the clinical validation of our microarray data. Meanwhile, we have identified predominant pathways of target genes associated with the selected circRNAs in KEGG pathway analysis. Among the top 10 most enriched pathways, Wnt signaling pathway is frequently associated with CRC^56^. Upregulated Wnt signaling can suppress checkpoint kinase 1 (CHK1) pathway in p53 wild-type CRC cells, as a novel mechanism for 5-FU resistance^57^. Hu *et al*. discovered that fibroblast exosomes activated the Wnt signaling pathway in CRC for development of cancer stem cells (CSCs) that are responsible for drug resistance^58^. Our findings suggest that exosomal circRNAs may be involved in different signaling pathways to modulate chemoresistance in CRC. Further investigation is essential to study the interaction between exosomal circRNAs and signaling pathways in FOLFOX-resistant CRC.

Previous studies have demonstrated that exosomes can transfer drug resistance between cancer cells^59^. Our study has performed exosomes uptake assay and co-culture assay to demonstrate that exosomes can transfer chemo-resistance from FOLFOX-resistant HCT116-R cells into parental HCT116-P cells. We have selected PKH26 dye for exosomes staining in the uptake assay, due to its common reliability, stability and well-established protocols in previous studies^60–62^. We have visualized the uptake of PKH26-stained exosomes into the cytoplasm of HCT116-P cells upon co-culture. Our co-cultured HCT116-P cells have significantly higher viability in 5-FU as compared to control cells. Exosomes have been reported as regulators of chemo-resistance in CRC^58,63,64^. Exosomes derived from cetuximab-resistant RKO CRC cells can induce cetuximab resistance in drug-sensitive Caco-2 cells via downregulation of phosphatase and tensin homolog (PTEN) and activation of phosphorylated Akt^63^. Exosomes have been shown to transfer nucleic acids from drug-resistant cancer cells into drug-sensitive cells to expand the resistance capacity in cancers. Exosomes derived from carcinoma-associated fibroblasts transfer lncRNA H19 into CRC cells to enhance oxaliplatin-resistance^64^. In gastric cancer, exosomes have been reported to transfer miR-155-5p into recipient cells to confer paclitaxel-resistance^65^. Our qRT-PCR analysis has shown the upregulation of circ_0032883 and circ_0002039 in the HCT116-P cells upon co-cultured with HCT116-R exosomes. This suggests that CRC exosomes perform the selective transfer of circRNAs from drug-resistant cancer cells into drug-sensitive counterparts to modulate chemo-resistance in CRC.

We have selected hsa_circ_0000338 for functional analysis to elucidate its possible role in chemoresistance. Hsa_circ_0000338 is a circRNA derived from gene FCHSD2 that encodes for a protein involved in endocytosis and membrane receptor internalization^66^. Genome-wide association studies identified FCHSD2 as a novel susceptibility gene for Crohn’s disease in Korean and Japanese populations^67,68^. We performed siRNA-knockdown of hsa_circ_0000338 in HCT116-R cell line, which improved drug resistance. This implicates that hsa_circ_0000338 may exhibit tumor suppressive properties to sensitize CRC cells towards drug treatment. However, hsa_circ_0000338 was selectively transferred via HCT116-R exosomes into co-cultured HCT116-P cells, which showed higher viability towards drug treatment as compared to control cells. This suggests that hsa_circ_0000338 may play an oncogenic role in HCT116-R exosomes to enhance drug resistance of recipient cells. Therefore, we have proposed that hsa_circ_0000338 may exhibit dual regulatory roles in chemo-resistant CRC, with different properties in CRC cells (tumor-suppressive) and CRC exosomes (oncogenic). High level of hsa_circ_0000338 in HCT116-R exosomes suggests a possible cellular mechanism of our HCT116-R cell lines to regulate intracellular expression of this circRNAs. CircRNAs were enriched in cancer-derived exosomes as compared to producer cells, to possibly regulate intracellular circRNAs levels^69^. Certain miRNAs in cancer-derived exosomes have been reported with a dual regulatory role in tumor progression, depending on parental cells and recipient cells^70^. Further in-depth analysis is required to elucidate the functions of hsa_circ_0000338 in FOLFOX-resistant CRC.

In conclusion, our study has presented the microarray profile of exosomal circRNAs in FOLFOX-resistant colon cancer cells. We have identified 105 upregulated and 34 downregulated circRNAs in exosomes from FOLFOX-resistant HCT116-R cell line. We have demonstrated that drug resistance can be transferred from resistant cells into sensitive cells via uptake of exosomes. Differentially upregulated hsa_circ_0000338 in exosomes could serve as a potential biomarker for early prediction of chemoresistance in CRC. Further investigation is essential to elucidate the possible roles of exosomal circRNAs in chemo-resistant CRC.

## Supplementary information

Supplementary
