# Supplementary material for: Extracellular Vesicle-derived circular RNAs confers chemoresistance in Colorectal cancer
Source: Sci Rep. 2019 Nov 11;9:16497. doi: 10.1038/s41598-019-53063-y (PMC6848089; doi:10.1038/s41598-019-53063-y)
Supplement: Supplementary file 1 — Supplementary [file 41598_2019_53063_MOESM1_ESM.docx]

**Extracellular Vesicle-derived circular RNAs confers chemoresistance in Colorectal cancer**

Kha Wai Hon^1^, Nurul Syakima Ab-Mutalib^1^, Nik Muhd Aslan Abdullah^2^, Rahman Jamal^1,^ Nadiah Abu^1^*

^1^UKM Medical Molecular Biology Institute (UMBI), Universiti Kebangsaan Malaysia

^2^Department of Oncology and Radiotherapy, UKM Medical Center, Universiti Kebangsaan Malaysia

*Correspondence to: Nadiah Abu (nadiah.abu@ppukm.ukm.edu.my)

UKM Medical Molecular Biology Institute (UMBI),

Universiti Kebangsaan Malaysia

**Supplementary Materials**

| Gender |  |
| --- | --- |
| Male | 14 |
| Female | 3 |
| Race |  |
| Malay | 12 |
| Chinese | 5 |
| Age |  |
| ≤ 50 | 5 |
| >50 | 12 |
|  |  |
| Tumour stage |  |
| II | 2 |
| III | 9 |
| IV | 6 |
| Chemo-response |  |
| Sensitive | 7 |
| Resistant | 10 |

Table S1: Demographic data of patients recruited for this study.


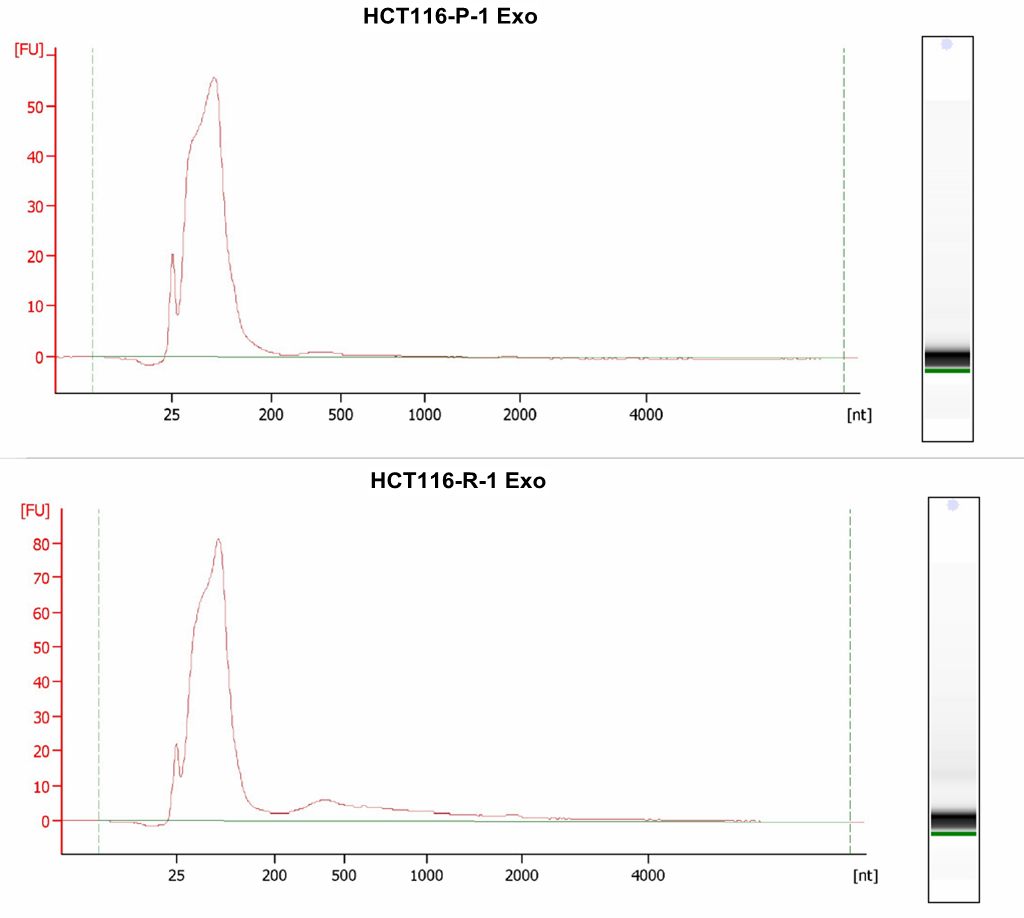


Figure S2: Representative images of Bioanalyzer results showing presence RNA in HCT116-P and HCT116-R exosomes. Exosomal RNA mainly contain small RNA with the absence of rRNA.

| **Primer Name** | **Sequence (Direction from 5' to 3')** |
| --- | --- |
| GAPDH Forward | GGATTTGGTCGTATTGGGC |
| GAPDH Reverse | TGGAAGATGGTGATGGGATT |
| L13 Forward | CGCTCACGCCTATAGTCTCA |
| L13 Reverse | AGATGCACACTCGTCTTCCA |
| hsa_circ_0000338 Forward | CCAAGCTGTGCAGAACACAT |
| hsa_circ_0000338 Reverse | CAAACGGGATACATGCTCCT |
| hsa_circ_0066629 Forward | CGGCCAAATCAGTGTTGTAA |
| hsa_circ_0066629 Reverse | CACAATGGCGAGGAATCTCT |
| hsa_circ_0032883 Forward | CACTGTGCAGACCATATCAGTTT |
| hsa_circ_0032883 Reverse | TCCCAACTTGTCCTGTTGCT |
| hsa_circ_0002039 Forward | GGAGGAGCACAAGAGCGTTA |
| hsa_circ_0002039 Reverse | CTGTGGTATCTGGGCGTTTT |

Table S3: All the primer sequences used for validation in this study.


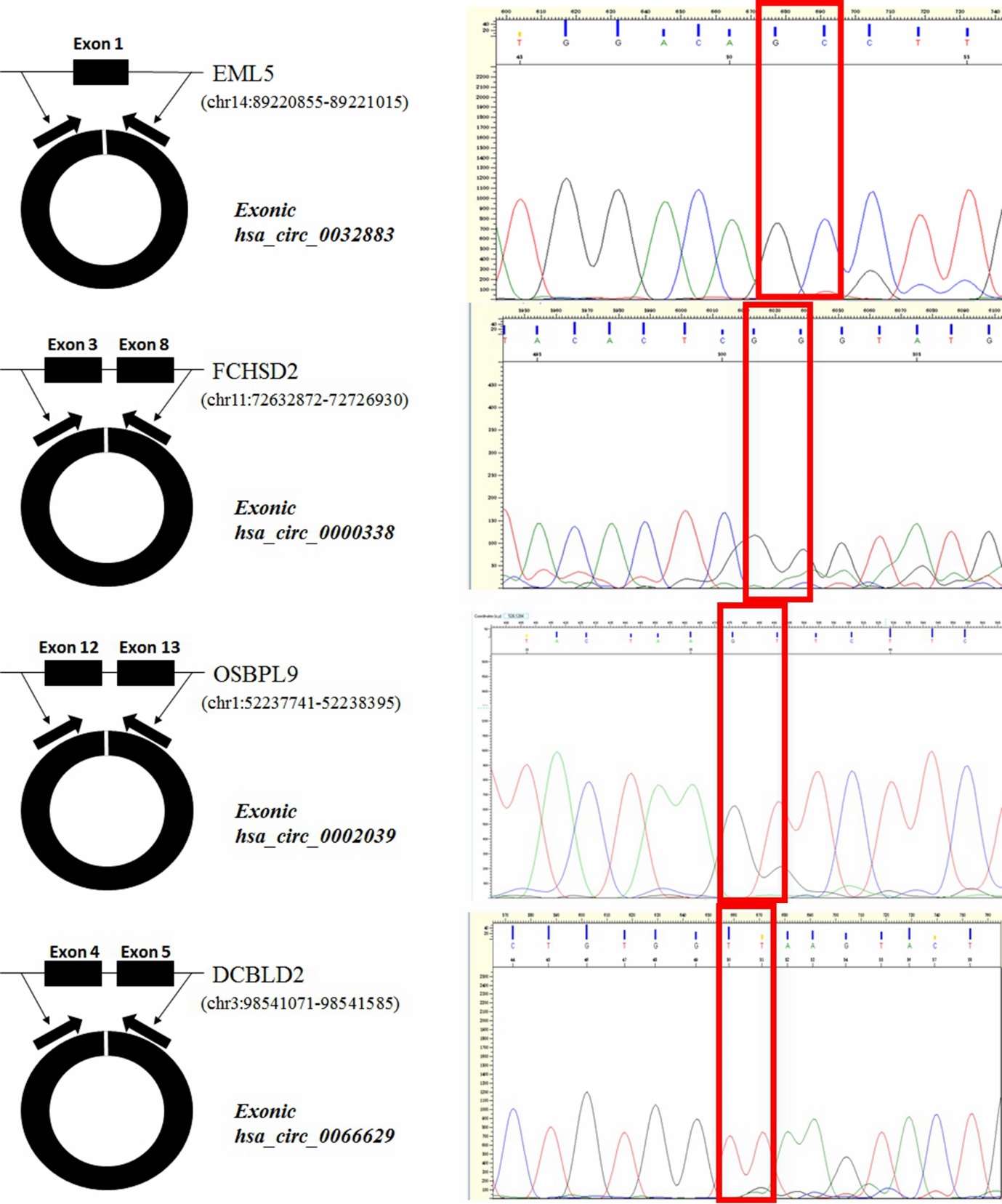


Figure S4: Sanger sequencing confirms the junction sequence for PCR product of divergent primers designed for each circRNA.


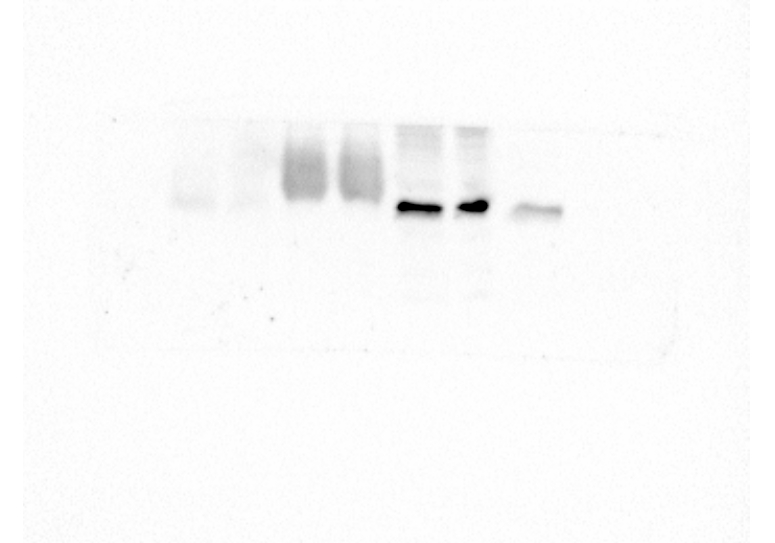


CD9


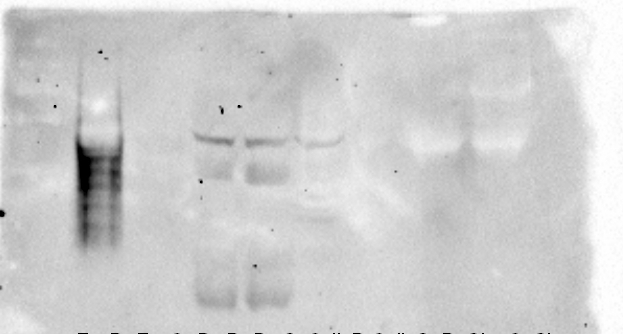


CD63


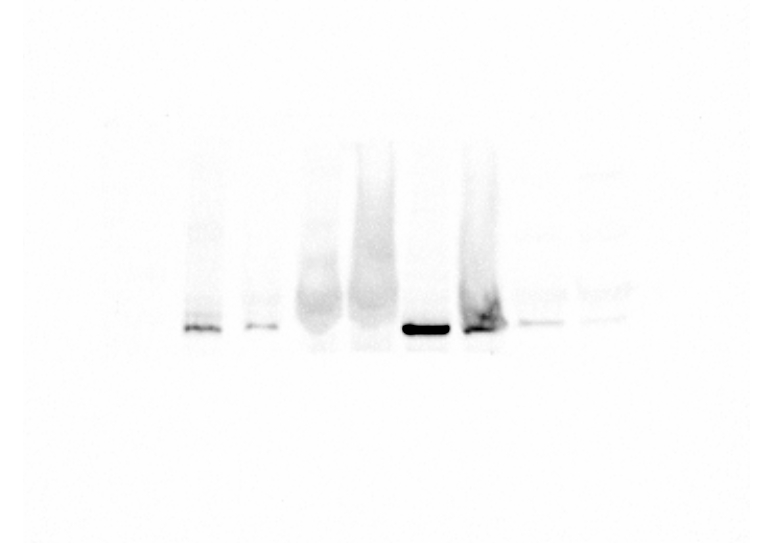


TSG101

Figure S5: Original blots for Western blot.

| CircRNA | CircBase ID | Fold Change | P-value |
| --- | --- | --- | --- |
| hsa_circRNA_103107 | hsa_circ_0061251 | 3.35 | 0.0388 |
| hsa_circRNA_100229 | hsa_circ_0002039 | 3.18 | 0.0066 |
| hsa_circRNA_405228 | - | 2.98 | 0.0389 |
| hsa_circRNA_407171 | - | 2.93 | 0.0079 |
| hsa_circRNA_061260 | hsa_circ_0061260 | 2.93 | 0.0311 |
| hsa_circRNA_104730 | hsa_circ_0002874 | 2.91 | 0.0018 |
| hsa_circRNA_072816 | hsa_circ_0072816 | 2.87 | 0.0126 |
| hsa_circRNA_048148 | hsa_circ_0048148 | 2.86 | 0.0119 |
| hsa_circRNA_402647 | - | 2.75 | 0.0212 |
| hsa_circRNA_404185 | - | 2.74 | 0.0280 |
| hsa_circRNA_008952 | hsa_circ_0008952 | 2.67 | 0.0211 |
| hsa_circRNA_005575 | hsa_circ_0005575 | 2.64 | 0.0011 |
| hsa_circRNA_401771 | - | 2.64 | 0.0022 |
| hsa_circRNA_100415 | hsa_circ_0015529 | 2.59 | 0.0132 |
| hsa_circRNA_101424 | hsa_circ_0032883 | 2.58 | 0.0020 |
| hsa_circRNA_002465 | hsa_circ_0002465 | 2.57 | 0.0286 |
| hsa_circRNA_031007 | hsa_circ_0031007 | 2.56 | 0.0313 |
| hsa_circRNA_102060 | hsa_circ_0007539 | 2.55 | 0.0165 |
| hsa_circRNA_404571 | - | 2.55 | 0.0049 |
| hsa_circRNA_103635 | hsa_circ_0069715 | 2.53 | 0.0264 |

Table S6: List of top 20 most differentially upregulated circRNAs in HCT116-R exosomes.
